# Localized Surface Plasmon Resonance in Metamaterials Composed of As_1−*z*_Sb_*z*_ Semimetal Nanoparticles in Al_*x*_Ga_1−*x*_As_1−*y*_Sb_*y*_ Semiconductor Matrix

**DOI:** 10.3390/nano13081355

**Published:** 2023-04-13

**Authors:** Vyacheslav M. Silkin, Sergey V. Eremeev, Vitalii I. Ushanov, Vladimir V. Chaldyshev

**Affiliations:** 1 Departamento de Polímeros y Materiales Avanzados: Física, Química y Tecnología, Facultad de Ciencias Químicas, Universidad del País Vasco (UPV-EHU), Apdo. 1072, E-20080 San Sebastián, Spain; 2 Donostia International Physics Center (DIPC), Paseo de Manuel Lardizabal 4, E-20018 San Sebastián, Spain; 3 IKERBASQUE, Basque Foundation for Science, E-48011 Bilbao, Spain; 4 Institute of Strength Physics and Materials Science, Siberian Branch, Russian Academy of Sciences, 634055 Tomsk, Russia; 5 Ioffe Institute, 26 Politekhnicheskaya Str., 194021 Saint Petersburg, Russia; ushanovvi@mail.ioffe.ru

**Keywords:** metal–semiconductor metamaterials, nanoparticles, dielectric function, optical absorption, plasmon resonance

## Abstract

We analyze the possibility to realize a localized surface plasmon resonance in metamaterials composed of As${}_{1-z}$Sb${}_{z}$ nanoparticles embedded in an Al${}_{x}$Ga${}_{1-x}$As${}_{1-y}$Sb${}_{y}$ semiconductor matrix. To this end, we perform ab initio calculations of the dielectric function of the As${}_{1-z}$Sb${}_{z}$ materials. Changing the chemical composition *z*, we trace the evolution of the band structure, dielectric function, and loss function. In terms of the Mie theory, we calculate the polarizability and optical extinction of a system of As${}_{1-z}$Sb${}_{z}$ nanoparticles in an Al${}_{x}$Ga${}_{1-x}$As${}_{1-y}$Sb${}_{y}$ environment. We show a possibility to provide localized surface plasmon resonance near the band gap of the Al${}_{x}$Ga${}_{1-x}$As${}_{1-y}$Sb${}_{y}$ semiconductor matrix by a built-in system of As${}_{1-z}$Sb${}_{z}$ nanoparticles strongly enriched by Sb. The results of our calculations are supported by available experimental data.

## 1. Introduction

Localized surface plasmons (LSPs)—collective excitations of valence electrons in metallic nanoparticles (NPs)—can resonantly enhance optical fields at the sub-wavelength scale in the medium in which the NPs are embedded [1]. The resonance condition depends on the NPs’ size, shape, and concentration and the dielectric properties of both the NPs and medium. If an NP is small enough that the retardation effects are negligible and it has a spherical shape, the condition of the LSP resonance (LSPR) reads

(1) Re[ϵ+2ϵm]=0,

where the real part of the dielectric permittivity ϵ of the NP is negative due to LSPs, and the real part of the permittivity $\epsilon_{m}$ of the dielectric medium, in which the NP is embedded, is positive.

Composite optical metamaterials supporting LSPR have been utilized since ancient times to produce beautiful and stable colored glasses [1]. Nowadays, the related research and development are focused on a wide variety of applications in physics, chemistry, optics, biology, medicine, etc., whenever an enhancement in light–matter interactions is essential [2,3]. In particular, systems of plasmonic NPs embedded in a semiconductor matrix can improve light harvesting in solar cells [4], create exceptional linear and nonlinear optical properties [5], and provide ultra-fast operations [6]. Nanoscale plasmonic metamaterials can be used to modify photon-matter interactions in a way that is essential for quantum information technology, which requires fast operations with qubits [7].

It is emergent to create plasmonic NPs in semiconductor materials, which could provide LSPR in the optical frequency range near the band gap of the semiconductor. However, the problem cannot be solved by local doping of the semiconducting matrix itself. The frequency range achievable in this way is limited by several THz due to the relatively low density of thus-created free electrons [2]. Thus, metal NPs are required to reach this goal.

Silver and gold are materials of choice for many applications in nanoplasmonics. Unfortunately, the fabrication technology of Ag or Au NPs is not compatible with molecular-beam epitaxy (MBE) or vapor-phase epitaxy (VPE), which are the common growth technologies for the production of III-V semiconductor materials and devices, such as solar cells, lasers, light-emitting diodes, etc. A system of Ag or Au plasmonic NPs can be produced on the surface of the semiconductor nanostructure by post-growth deposition and treatments [8,9], but it is extremely hard to bury such NPs into the bulk of an epitaxial film or heterostructure. Some atomic components of the III-V semiconductor compounds, such as metallic or semimetallic Al, In, Ga, As, and Sb, are widely available in MBE setups. There were extensive efforts to create NPs of these materials in the bulk of a semiconducting matrix by variation of the epitaxial growth conditions. It was found that alloys of group III elements, including indium, gallium, and aluminum, can be obtained as droplets on the surface of III-V epitaxial films in the growth process [10,11,12], but incorporation of such droplets in the bulk is problematic.

An appropriate technology was developed for the formation of As and As${}_{1-z}$Sb${}_{z}$ NPs in the bulk of GaAs and Al${}_{x}$Ga${}_{1-x}$As${}_{1-y}$Sb${}_{y}$ epitaxial films by MBE [13,14,15,16]. The procedure consists of two steps. In the first step, an epitaxial layer is grown by MBE at low temperature (LT), typically 150–250 °C, under As-rich conditions. These growth conditions provide the incorporation of a large amount of the excess arsenic in the crystalline lattice, mostly as arsenic antisite defects, As${}_{\mathrm{Ga}}$ [17,18]. In the second step, the LT-grown film is annealed at a high temperature under As over-pressure, or it can be overgrown by other Al${}_{x}$Ga${}_{1-x}$As${}_{1-y}$Sb${}_{y}$ layers at a certain temperature, typically 500–700 °C, and conditions can be optimized for the best crystalline quality of these layers. In the second step, As or As${}_{1-z}$Sb${}_{z}$ nanoparticles in the bulk of the LT-grown layer are formed as a result of the thermally-activated migration of the non-stoichiometry-related point defects [19,20,21]. Despite the formation of NPs, the matrix always has a zincblende crystalline structure and shows a band gap that is variable from 1.4 eV to 2.5 eV according to the chemical composition of the Al${}_{x}$Ga${}_{1-x}$As${}_{1-y}$Sb${}_{y}$ solid solution [22]. It should be noted that in the Sb-free case, the NPs forming in LT-grown Al${}_{x}$Ga${}_{1-x}$As are composed of pure arsenic [23,24]. However, quite a small Sb concentration (y≈0.03) in the LT-grown Al${}_{x}$Ga${}_{1-x}$As${}_{1-y}$Sb${}_{y}$ results in the formation of NPs with an Sb concentration z≥0.9 [25].

Systems of arsenic NPs in a GaAs matrix, being well documented by transmission electron microscopy, do not show any notable features in the near-infrared optical spectra [13,26]. The As–Al${}_{x}$Ga${}_{1-x}$As metamaterial was found to be optically transparent for the photons with energy below the Al${}_{x}$Ga${}_{1-x}$As band gap when the formation of the system of the As NPs was completed and the concentration of optically active point defects was reduced to a rather low equilibrium value. In contrast, the As${}_{1-z}$Sb${}_{z}$–Al${}_{x}$Ga${}_{1-x}$As${}_{1-y}$Sb${}_{y}$ metamaterials with the same band gap of the matrix and similar geometrical parameters of the built-in NPs (size, shape, concentration, and spatial distribution) exhibit substantial optical extinction in the infrared optical range [27,28,29]. This extinction becomes experimentally remarkable at photon energies above 1 eV and increase until reaching the band edge of the semiconductor matrix [30]. The observed optical extinction was attributed to the LSPR in the system of As${}_{1-z}$Sb${}_{z}$ NPs embedded in the Al${}_{x}$Ga${}_{1-x}$As${}_{1-y}$Sb${}_{y}$ matrix [27,28]. The corresponding extinction spectra were phenomenologically described by using a Drude dielectric function for the As${}_{1-z}$Sb${}_{z}$ NPs and the known dielectric permittivity of the Al${}_{x}$Ga${}_{1-x}$As${}_{1-y}$Sb${}_{y}$ matrix [31].

The physical rationale of the LSPR in the As${}_{1-z}$Sb${}_{z}$–Al${}_{x}$Ga${}_{1-x}$As${}_{1-y}$Sb${}_{y}$ metamaterials and the absence of such a resonance in As–Al${}_{x}$Ga${}_{1-x}$As metamaterials cannot be provided in terms of the phenomenological Drude model since the parameters of this model and its applicability to As and As${}_{1-z}$Sb${}_{z}$ NPs have not been justified. To the best of our knowledge, data on the dielectric permittivity of the arsenic and antimony–arsenic alloy are not available. Such data for pure antimony are limited [32,33,34] and have to be verified. Therefore, it is emergent to obtain the dielectric properties of As, Sb, and As${}_{1-z}$Sb${}_{z}$ alloys by ab initio computations along with the band structure of these materials over the whole Brillouin zone in the vicinity of the Fermi level.

In this paper, we present the results of a systematic study of the dielectric properties of As${}_{1-z}$Sb${}_{z}$ compounds with variable *z* concentration. For systems of As${}_{1-z}$Sb${}_{z}$ NPs embedded in an Al${}_{x}$Ga${}_{1-x}$As${}_{1-y}$Sb${}_{y}$ semiconductor matrix, we calculate the optical extinction spectra in terms of the Mie theory. We show that the LSPR condition (1) cannot be satisfied in As-Al${}_{x}$Ga${}_{1-x}$As metamaterials, whereas systems of antimony-rich NPs should provide quite a strong optical extinction in the near-infrared optical range. The calculated extinction spectra appeared to be consistent with our experimental observations.

The rest of the paper is organized as follows. In Section 2, the computational details for ab initio calculations of the atomic structure, the electronic band structure, and the dielectric function at small momentum transfers are described. The band structure and dielectric function of As${}_{1-z}$Sb${}_{z}$ compounds evaluated at four values of *z* are discussed in Section 3. In Section 4, we present the optical extinction coefficients in As${}_{1-z}$Sb${}_{z}$–Al${}_{x}$Ga${}_{1-x}$As${}_{1-y}$Sb${}_{y}$ metamaterials obtained on the base of these dielectric functions and compare them with experimental data. The main results are summarized in Section 5 along with the concluding remarks. Unless otherwise stated, atomic units (ℏ = *e* = $m_{e}$ = 1) are used throughout.

## 2. Calculation Methods and Computational Details

The electronic structure was calculated using the density functional theory formalism simulating the electron–ion interactions with the Troullier–Martins norm-conserving pseudopotentials [35]. The Ceperley–Alder functional [36] in the Perdew–Zunger parametrization [37] was employed for the exchange-correlation potential. The plane-wave basis set with an energy cutoff of 540 eV was used for all systems. The self-consistent band structure calculations were realized with a custom code [38].

**Figure 1:**
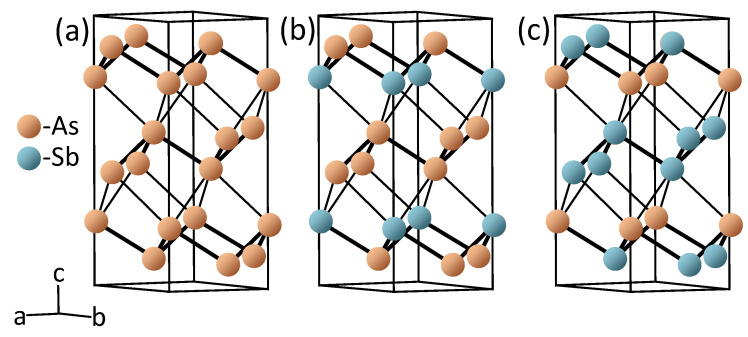
Crystal lattices employed in the calculations for (**a**) As, (**b**) As${}_{0.67}$Sb${}_{0.33}$, and (**c**) As${}_{0.33}$Sb${}_{0.67}$. The lattice for pure Sb is similar to that in (**a**).

The As${}_{z}$Sb${}_{1-z}$ crystal lattices were simulated with 6-atom supercells consisting of 6×(1−z) As and 6×z Sb atoms, as shown in Figure 1. The calculations were realized considering four systems with *z* = 0, 0.25, 0.5, and 1. The lattice parameters and the atomic positions were simultaneously relaxed in each system until the residual forces were less than 0.005 eV/Å. The structural optimization was realized with the Vienna ab initio simulation package [39,40], with core electrons represented by projector augmented wave (PAW) potentials [41]. The kinetic cutoff energy was 400 eV and a 12 × 12 × 6 Monkhorst–Pack *k*-point mesh [42] was used to sample the Brillouin zone (BZ).

The frequency-dependent dielectric functions were calculated in the framework of time-dependent density functional theory (TD-DFT) [43,44]. The macroscopic dielectric function $\epsilon_{M}$ probed in the optical experiments is given by $\epsilon_{M}\left(q,\omega \right)={\left[1/\epsilon_{GG^{\prime }}^{-1}\left(q,\omega \right)\right]}_{G=0G^{\prime }=0}$, where Gs are the reciprocal-lattice vectors, **q** is the small momentum transfer, and ω is the transferred energy. The inverse dielectric matrix $\epsilon_{GG^{\prime }}^{-1}\left(q,\omega \right)$ is related to the density response function of interacting electrons $\chi_{GG^{\prime }}\left(q,\omega \right)$ by

(2) $$\epsilon_{GG^{\prime }}^{-1}\left(q,\omega \right)=\delta_{GG^{\prime }}+\chi_{GG^{\prime }}\left(q,\omega \right)V_{G^{\prime }}\left(q\right),$$

where $\delta_{GG^{\prime }}$ is the unity matrix and $V_{G^{\prime }}\left(q\right)=4\pi /{\left|q+G^{\prime }\right|}^{2}$ is the Fourier transform of a bare Coulomb potential. The matrix $\chi_{GG^{\prime }}\left(q,\omega \right)$ can be obtained from the matrix equation

(3) $$\chi_{GG^{\prime }}\left(q,\omega \right)=\chi_{GG^{\prime }}^{o}\left(q,\omega \right)+\sum_{G_{1},G_{2}}\chi_{GG_{1}}^{o}\left(q,\omega \right)\left[V_{G_{1}}\delta_{G_{1}G_{2}}+K_{G_{1}G_{2}}^{\mathrm{xc}}\left(q,\omega \right)\right]\chi_{G_{2}G^{\prime }}\left(q,\omega \right).$$

Here, the kernel $K^{\mathrm{xc}}$ accounts for the exchange-correlation effects. In the present work, we checked how the results depend on its shape considering two approximations: a so-called random-phase approximation (RPA) (when $K^{\mathrm{xc}}$ is set to zero) and an adiabatic local density approximation (ALDA) [43,45].

In Equation (3) $\chi_{GG^{\prime }}^{o}\left(q,\omega \right)$ is the response function of the non-interacting Kohn–Sham electrons, defined as

(4) $$\begin{array}{ccc} \chi_{GG^{\prime }}^{o}\left(q,\omega \right) & = & \frac{2}{\Omega }\sum_{k}^{\mathrm{BZ}}\sum_{n}^{\mathrm{occ}}\sum_{n^{\prime }}^{\mathrm{unocc}}\frac{f_{nk}-f_{n^{\prime }k+q}}{\varepsilon_{nk}-\varepsilon_{n^{\prime }k+q}+\left(\omega +i\eta \right)}\\ & \times & \langle \psi_{nk}|e^{-i(q+G)r}|\psi_{n^{\prime }k+q}\rangle \langle \psi_{n^{\prime }k+q}|e^{i(q+G^{\prime })r}|\psi_{nk}\rangle . \end{array}$$

Here, 2 accounts for spin, Ω is the unit cell volume, $f_{nsk}$ is the Fermi occupation number at a temperature of zero, and η is infinitesimal. Summation over the BZ is realized on a 48×48×18k mesh. All valence one-particle states with energies $\varepsilon_{nk}$ and wavefunctions $\psi_{nk}$ up to an energy of 50 above the Fermi level were taken into account. In the expansion of matrices $\chi^{o}$, χ, $\epsilon^{-1}$, and ϵ, 100 G vectors were included. Some other calculation details can be found elsewhere [46].

## 3. Calculation Results and Discussion

**Figure 2:**
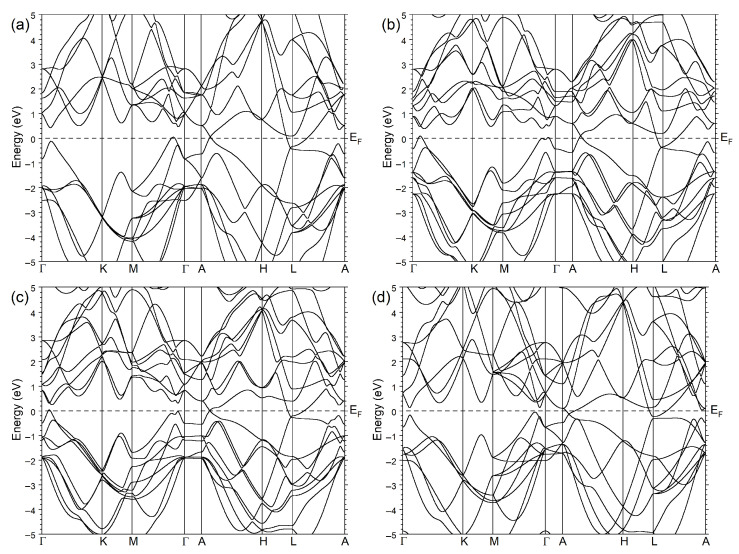
Electronic structure of (**a**) As, (**b**) As${}_{0.67}$Sb${}_{0.33}$, (**c**) As${}_{0.33}$Sb${}_{0.67}$, and (**d**) Sb. The Fermi level position, $E_{F}$, which is set to zero, is marked by horizontal dashed line.

The calculated electronic structure for all four systems is presented in Figure 2. Overall, they are very similar in the number of energy bands and their dispersion in this energy interval. The main effect of the change in the atomic composition consists of the energy splitting of the bands in As${}_{1-z}$Sb${}_{z}$ with non-integer *z*s due to reducing symmetry. Additionally, only two bands (in As and Sb, these bands are generated in the Γ point vicinity due to the unit cell choice) are located in a close vicinity to the Fermi level, resulting in the semimetallic behaviour. Nevertheless, some quantitative distinctive features caused by differences in the ionic pseudopotentials of As and Sb and in the lattice parameters can be noted. The double degenerate energy band in the Γ point vicinity splits into two bands in the systems with a fractional Sb composition. Additionally, in As, the Dirac point (DP) in the Dirac cone observed along the AH direction is located slightly above $E_{F}$. In As${}_{0.67}$Sb${}_{0.33}$, it becomes more depopulated. When the Sb concentration increases up to 0.67, the DP is placed exactly at zero energy. In pure Sb, the DP drops below the Fermi level. The energy band crossing the Fermi level at ΓM in As has two maxima in As${}_{0.67}$Sb${}_{0.33}$ and As${}_{0.33}$Sb${}_{0.67}$. In the former case, these maxima reach the Fermi level, whereas in the latter case, this part locates entirely below it. In Sb, this part of the band again has one maximum and is completely occupied. The topology of the Fermi surface in the Γ point vicinity also changes with the change in the composition. In As and Sb, along ΓK, the band closest to the Fermi level is totally below the Fermi level. On the contrary, in As${}_{0.67}$Sb${}_{0.33}$ and As${}_{0.33}$Sb${}_{0.67}$, this band crosses $E_{F}$. The bands that provide a major contribution to the density of states at the Fermi level are located in a vicinity of L point. One can see that, in As${}_{0.67}$Sb${}_{0.33}$, near the L point, the two upper valence bands are located slightly closer to $E_{F}$ in comparison to As. With the Sb concentration increasing up to 0.67, they approach the Fermi level even more. In pure Sb, these bands are shifted slightly downward.

**Figure 3:**
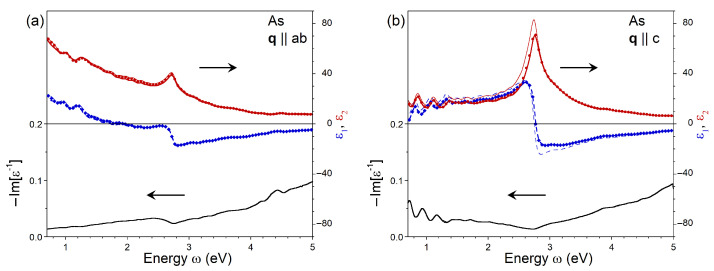
Imaginary (red dashed line) and real (blue solid line) parts of dielectric function in As calculated at q pointing in (**a**) ab plane and (**b**) *c* direction obtained with inclusion of the local-field effects and the RPA kernel. The respective loss function, −Im$\left[\epsilon^{-1}\right]$, is shown by black solid line. Imaginary and real parts of the RPA dielectric function evaluated without inclusion of local-field effects are shown by thin red dashed and thin blue solid lines, respectively. The parts of dielectric function obtained with inclusion of both the local-field effects and ALDA kernel are shown by filled diamonds and circles.

In Figure 3, we plot the real $\epsilon_{1}$ and imaginary $\epsilon_{2}$ parts of the dielectric function of As, as well as the energy-loss function, −Im$\left[\epsilon^{-1}\right]$, at small qs pointing in (a) the ab plane and (b) the direction *c*. One can notice that the dielectric function in As presents notable anisotropy in this energy range. Whereas in Figure 3b, $\epsilon_{2}$ is dominated by a strong peak centered at an energy of 2.75 eV, in Figure 3a, a similar peak is significantly less intense and is located at 2.70 eV. Additionally, on the low-energy side, the value of $\epsilon_{2}$ is up to four times larger in Figure 3a in comparison to Figure 3b. These differences in $\epsilon_{2}$ produce notable variations in the respective real parts. Thus, when q||ab, $\epsilon_{1}$ becomes negative for the energies above ≈1.8 eV (Figure 3a), whereas $\epsilon_{1}$ changes its sign for q∥c at an energy near 2.75 eV (Figure 3b). The shallow minima reaching −17.5 (−17) are observed in $\epsilon_{1}$ at an energy of ≈2.85 eV (≈2.8 eV) for the ab (*c*) polarization.

Comparing the dielectric functions in Figure 3b obtained for q along the *c* axis without and with the LFEs included, one can see that the LFEs slightly change the amplitude and keep the energy position of the prominent interband 2.75 eV peak of $\epsilon_{2}$. As a result, $\epsilon_{1}$ exhibits small variations in the nearby energy range as well. As for the data presented in Figure 3a, the LFEs have a negligible impact on ϵ evaluated at q with ab-plane polarization. The comparison of the data obtained by taking into account the RPA and the ALDA approximations for the $K^{\mathrm{xc}}$ kernel confirms the negligible role played by the exchange-correlations in the determination of the dielectric function in this material. Since similar effects are observed in all other systems of interest here, below, we will only report and discuss the data obtained with the inclusion of the LFEs and the ALDA kernel.

**Figure 4:**
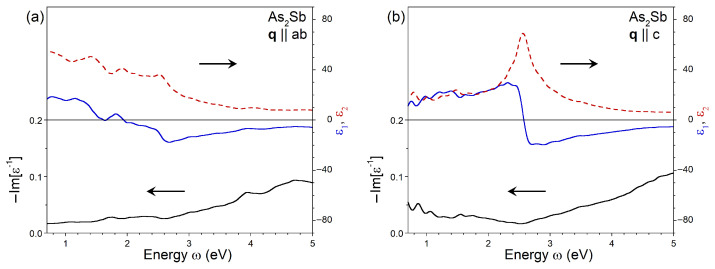
Imaginary (red dashed line) and real (blue solid line) parts of dielectric function in As${}_{0.67}$Sb${}_{0.33}$ calculated at q pointing in (**a**) ab plane and (**b**) *c* direction obtained with inclusion of the local-field effects and the ALDA kernel. The respective loss function, −Im$\left[\epsilon^{-1}\right]$, is shown by black solid line.

The substitution of 33% of the As atoms in the crystal lattice by Sb atoms results in a notable variation of the dielectric function for q oriented in the ab plane, as seen in Figure 4a. The dominant peak at 2.7 eV presented in $\epsilon_{2}$ of pure As is downward-shifted to 2.5 eV in As${}_{0.67}$Sb${}_{0.33}$, and its magnitude is strongly suppressed. Additionally, in As${}_{0.67}$Sb${}_{0.33}$, a broad prominent peak emerges at an energy of 1.4 eV. Consequently, the real part of the dielectric function varies near zero at remarkably low energies for q oriented in the ab plane. The in-plane component of $\epsilon_{1}$ touches zero at 1.65 eV and crosses the zero line definitely at 1.93 eV. These changes in $\epsilon_{1}$ are accompanied by some decrease in its minimum value from −17 (located at 2.80 eV) in As to −17.5 (at ω = 2.65 eV) in As${}_{0.67}$Sb${}_{0.33}$. In the case of q∥c, presented in Figure 4b, the dominating peak in $\epsilon_{2}$ for As${}_{0.67}$Sb${}_{0.33}$ is centered at ω = 2.55 eV, i.e., it is shifted downward by 0.2 eV in comparison with pure As. The same energy shift of the zero crossing occurs in $\epsilon_{1}$. For q∥c, the real part of ϵ has the lowest value of −19, and the substitution of 33% of As atoms by Sb atoms results in a decrease of $\epsilon_{1}$ at energies around 2.8 eV.

**Figure 5:**
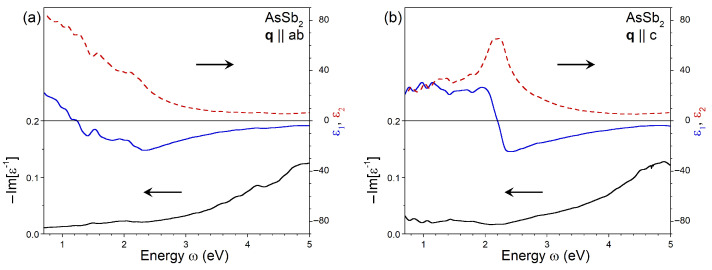
Imaginary (red dashed line) and real (blue solid line) parts of dielectric function in As${}_{0.33}$Sb${}_{0.67}$ calculated at q pointing in (**a**) ab plane and (**b**) *c* direction obtained with inclusion of the local-field effects and the ALDA kernel. The respective loss function, −Im$\left[\epsilon^{-1}\right]$, is shown by black solid line.

Further increases in the Sb concentration maintain the same tendencies. As seen in Figure 5a, in As${}_{0.33}$Sb${}_{0.67}$, the amplitude of $\epsilon_{2}$ at q∥ab is notably enhanced on the lower-energy side. This is accompanied by washing out almost all significant features in this energy range. In consequence, for this polarization, $\epsilon_{1}$ becomes negative at energies above 1.25 eV. Now, the shallow minimum of −23 in $\epsilon_{1}$ locates at an energy of 2.3 eV. In the case of dielectric function with polarization along the *c* crystal direction, plotted in Figure 5b, the amplitude of $\epsilon_{2}$ somehow increases at low energies in comparison to the As${}_{0.67}$Sb${}_{0.33}$ case. The dominant peak in $\epsilon_{2}$ becomes significantly wider, and its energy position shifts downward to 2.2 eV. The respective zero-crossing of the $\epsilon_{1}$ curve occurs at the same energy. The $\epsilon_{1}$ reaches a minimum value of −25 at ω = 2.4 eV.

**Figure 6:**
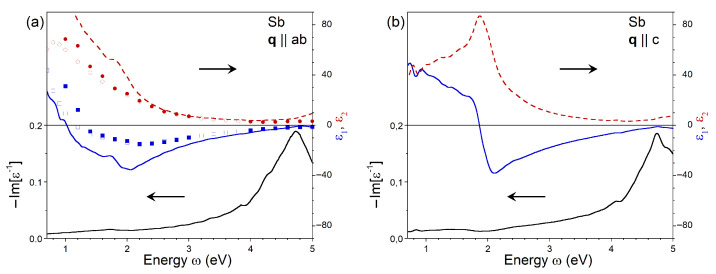
Imaginary (red dashed line) and real (blue solid line) parts of dielectric function in Sb calculated at q pointing in (**a**) ab plane and (**b**) *c* direction obtained with inclusion of the local-field effects and the ALDA kernel. The respective loss function, −Im$\left[\epsilon^{-1}\right]$, is shown by black solid line. The dielectric function components derived from the optical measurements are shown by filled [34] and open [32,33] symbols.

Figure 6 shows the calculated dielectric function for pure Sb along with available experimental data derived from optical measurements. In the low-energy region, the imaginary part of ϵ for q∥ab polarization is larger than those of pure As or the As${}_{z}$Sb${}_{1-z}$ alloy. It reaches a value of 85 at ω = 1.2 eV. However, the $\epsilon_{2}$ of pure Sb is smaller in the high-energy region (above ∼2 eV). Here, the $\epsilon_{2}$ spectrum is rather featureless after a hump at an energy of 1.8 eV. The real part of the dielectric function for this polarization is negative over a wide range ω > 1.2 eV. It is more negative when compared to As${}_{z}$Sb${}_{1-z}$ with z<1. In particular, the minimum value $\epsilon_{1}=-35$ is observed at an energy of 2.05 eV. Such variations of the ϵ lead to a significant modification of the loss function, where a clear plasmonic peak can be seen at an energy of 4.75 eV, whereas an overdamped plasmon can be detected at energies of about 5 eV for lower Sb concentrations. The dielectric function of Sb evaluated at q∥c is reported in Figure 6b. In the imaginary part of ϵ, a dominant peak is observed at an energy of 1.85 eV. On the low-energy side of this peak, the magnitude of $\epsilon_{2}$ exceeds that for pure As and As${}_{z}$Sb${}_{1-z}$, whereas in the higher-energy part, it is notably smaller. The presence of a well-defined peak in $\epsilon_{2}$ results in a sharp drop in $\epsilon_{1}$, which becomes negative at the same energy. The real part reaches a minimum of −38 at ω = 2.1 eV and gradually disperses upward upon an increase in the energy. At ω≈ 4.75 eV, it almost reaches zero. This fact, in combination with a small $\epsilon_{2}$ in this region, produces a well-defined plasmonic peak in the loss function at the same energy.

For comparison, using symbols, in Figure 6a, we plot the dielectric functions derived from optical experiments [32,33,34]. The experimental and theoretical imaginary parts of the dielectric function are very close to each other in the energy range above 2.4 eV. At lower energies, the theoretical values are considerably larger than the experimentally determined ones. Regarding the real part of ϵ, the experimental curves have a more shallow minimum, reaching at the bottom a value of −15, whereas the theoretical curve has a minimum at −35. Additionally, the left zero crossing in the experimental $\epsilon_{1}$ occurs at energies larger than the theoretical prediction. Nevertheless, the differences between the experimental and the theoretical $\epsilon_{1}$ at energies below ∼1.3 eV are of the same magnitude as the spread in the experimental data.

## 4. Optical Properties of Metamaterials Composed of As${}_{1-z}$Sb${}_{z}$ Nanoparticles in Al${}_{x}$Ga${}_{1-x}$As${}_{1-y}$Sb${}_{y}$ Matrix

It is known that optical properties of metamaterials depend both on the chemical composition and the structure at the micro- and nano-scale. The structures of the metamaterials of interest are well documented [13,14,15,16,25,29]. In homogeneous undoped GaAs and Al${}_{x}$Ga${}_{1-x}$As${}_{1-y}$Sb${}_{y}$ matrices, the As and As${}_{1-z}$Sb${}_{z}$ NPs are randomly dispersed over the whole bulk of the LT-grown film. The NPs exhibit an almost spherical shape. The mean diameter of the NPs in the ensemble depends on the growth temperature and post-growth heat treatment. Typically, the mean radius, *r*, ranges from 2 to 10 nm. The mean distance, *l*, between neighboring NPs varies from 3.5*r* to 8.5*r*.

The optical range of interest in this research is approximately λ=500–1000 nm, which corresponds to the photon energy near and below the band gap edge of the semiconductor matrix. In this range, the approximation of small particles λ≫r,l is justified, and interaction between NPs via secondary electromagnetic field can be neglected.

Modeling of the optical extinction of the As${}_{1-z}$Sb${}_{z}$–Al${}_{x}$Ga${}_{1-x}$As${}_{1-y}$Sb${}_{y}$ metamaterials is performed in terms of the Mie theory for a system of spherical NPs randomly distributed in a bulk of a semiconductor matrix [47]. The extinction cross-section for a single NP reads

(5) $$C_{ext}=\frac{2\pi }{k^{2}}\sum_{i=1}^{\infty }\left(2i+1\right)Re\left(a_{i}+b_{i}\right),$$

where *k* is the light wavenumber in the matrix and $a_{i}$, $b_{i}$ are the scattering coefficients. Assuming the same magnetic permeability of the NP and matrix materials, the scattering coefficients can be calculated as follows [47]:

(6) $$a_{i}=\frac{m\psi_{i}\left(ms\right)\psi_{i}^{\prime }\left(s\right)-\psi_{i}\left(s\right)\psi_{i}^{\prime }\left(ms\right)}{m\psi_{i}\left(ms\right)\xi_{i}^{\prime }\left(s\right)-\xi_{i}\left(s\right)\psi_{i}^{\prime }\left(ms\right)},$$

(7) $$b_{i}=\frac{\psi_{i}\left(ms\right)\psi_{i}^{\prime }\left(s\right)-m\psi_{i}\left(s\right)\psi_{i}^{\prime }\left(ms\right)}{\psi_{i}\left(ms\right)\xi_{i}^{\prime }\left(s\right)-m\xi_{i}\left(s\right)\psi_{i}^{\prime }\left(ms\right)},$$

where $m^{2}=\epsilon /\epsilon_{m}$ is the ratio of the dielectric permittivities of the NP and surrounding matrix, s=kr, and $\psi_{i}\left(\rho \right)\;\mathrm{and}\;\xi_{i}\left(\rho \right)$ are the Riccati–Bessel functions. The resulting extinction coefficient, α, can be determined as a sum of contributions of all the NPs defined by Equations (5)–(7).

For small NPs, r≪λ, the Mie series can be reduced to the electric dipole approximation, in which absorption makes a dominant contribution to the optical extinction

(8) $$C_{ext}\approx C_{abs}=\frac{24\pi^{2}r^{3}\epsilon_{m}^{3/2}}{\lambda }\frac{\epsilon_{2}}{|\epsilon +2\epsilon_{m}{|}^{2}}.$$

In this case, the extinction coefficient does not depend on the NP size distribution. It is determined by the volume fraction, *f*, occupied by all NPs in the metamaterial.

(9) $$\alpha =\frac{3f}{4\pi r^{3}}C_{ext}.$$

In our numerical calculations, we utilized structural parameters of the As${}_{1-z}$Sb${}_{z}$–Al${}_{x}$Ga${}_{1-x}$As${}_{1-y}$Sb${}_{y}$ and As–Al${}_{x}$Ga${}_{1-x}$As metamaterials documented in Ref. [29]. The dielectric properties of the matrix were modeled using the extended Adachi formalism [48] for the Al${}_{x}$Ga${}_{1-x}$As ternary alloy. The Al concentration in the matrix was x≈0.6. The dielectric function of the NPs was taken from the calculations presented in the Section 3. The NPs were considered spheres with an average diameter of 6 nm. The filling factor *f* was used as the only fitting parameter. In our calculations, in terms of the Mie theory, we assumed that the system of nanoparticles is diluted. This assumption was based on experimental observations [29]. In the experiment, the volume occupied by NPs was as low as 0.003. For such a dilute system, the NPs are not in the near field of each other, and, consequently, the effect of secondary field induced by neighbors is small [47]. Therefore, the resultant optical spectra were calculated as additive contributions of all the NPs. It is also important that the fabrication technology prevents the formation of closely spaced pairs of NPs with a narrow gap between them [14,15]. Therefore, we do not consider the effects of electric hot spots.

**Figure 7:**
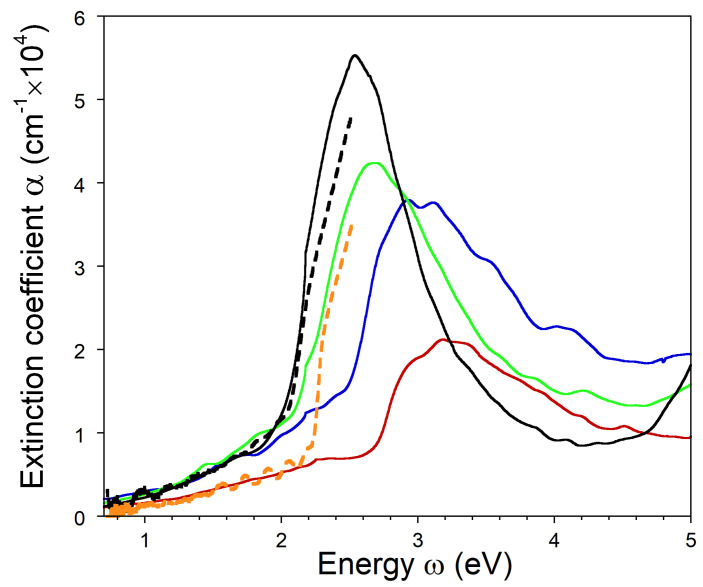
Calculated extinction coefficients for As${}_{1-z}$Sb${}_{z}$–Al${}_{x}$Ga${}_{1-x}$As${}_{1-y}$Sb${}_{y}$ and As–Al${}_{x}$Ga${}_{1-x}$As metamaterials consisting of Sb, As${}_{0.33}$Sb${}_{0.67}$, As${}_{0.67}$Sb${}_{0.33}$, and As NPs embedded in the semiconductor matrices (solid black, green, blue, and red lines, respectively). The experimentally measured extinction coefficients in As${}_{1-z}$Sb${}_{z}$–Al${}_{x}$Ga${}_{1-x}$As${}_{1-y}$Sb${}_{y}$ and As–Al${}_{x}$Ga${}_{1-x}$As metamaterials are shown by dashed black and orange lines, respectively.

The calculated spectra of the optical extinction coefficient are plotted in Figure 7 for the As${}_{1-z}$Sb${}_{z}$–Al${}_{x}$Ga${}_{1-x}$As${}_{1-y}$Sb${}_{y}$ metamaterials with different chemical compositions of As${}_{1-z}$Sb${}_{z}$ NPs and for the As–Al${}_{x}$Ga${}_{1-x}$As metamaterial. Figure 7 also shows experimental optical extinction spectra for these metamaterials recorded at room temperature. The latter were obtained from the experimental transmission, *T*, and reflection, *R*, spectra utilizing the Beer–Lamber law by the following expression:

(10) $$\alpha =-\ln\left(\frac{T}{1-R}\right).$$

The details of the optical and structural investigations are described in Ref. [29].

The calculated optical extinction spectra exhibit strong peaks originating from LSPR in the metamaterials. For the NPs consisting of pure As, the energy of LSPR is about 3.25 eV, and the respective peak has a width of about 1.1 eV. When the Sb concentration increases, the LSPR energy shows a substantial red shift. For the As${}_{0.67}$Sb${}_{0.33}$ NPs, the LSPR occurs at 3.05 eV and has approximately the same width. For the As${}_{0.33}$Sb${}_{0.67}$ NPs, the LSPR is predicted at 2.65 eV and has a width of 0.9 eV. Finally, for a system of pure Sb NPs, the LSPR energy is 2.55 eV, and the width of the extinction peak is 0.8 eV.

It is noticeable that the optical extinction spectra calculated for the Sb-rich NPs are well consistent with the experiment. The corresponding filling factor f=0.015 is reasonable; however, it is larger than the experimentally determined value of 0.004. The results of the calculations strongly support the experimental observation that systems of Sb-rich NPs provide a substantial optical extinction, whereas similar systems of As NPs are almost optically inactive. The physical reason for this phenomenon is that pure arsenic possesses a positive real part of the dielectric function at energies below 2.8 eV (see Figure 3), and, consequently, As NPs cannot support LSPR in any dielectric medium for wavelengths longer than 440 nm. The Sb-rich NPs exhibit plasmonic properties until much lower energies of 2.3 eV (As${}_{0.33}$Sb${}_{0.67}$) and 2.0 eV (Sb), as illustrated by Figure 5 and Figure 6. Structural investigations of the As${}_{1-z}$Sb${}_{z}$ NPs formed in Al${}_{x}$Ga${}_{1-x}$As${}_{1-y}$Sb${}_{y}$ by the technology described in Section 1 show a strong enrichment of the NPs by Sb. For a Sb concentration y≈0.03 in the matrix, its concentration in the NPs is z≥0.9 [25].

Both the calculations and experiments show that the LSPR is feasible in metamaterials formed by systems of Sb-rich nanoinclusions in the matrix of a III-V semiconductor compound with sufficiently large band gap. The plasmon resonance is commonly associated with high losses, which originate from the metallic portion of the metamaterial structure. Figure 7 demonstrates that the LSPR is well defined, with a remarkably large width of about 1 eV. The width and the strength of the resonance are determined by the imaginary part of the dielectric function. Under the resonant condition defined by Equation (1), the contribution of the real part of the dielectric function to the denominators in Equations (5)–(8) is zero, and the cross-section and coefficient of the optical extinction are inversely proportional to $\epsilon_{2}$. From this point of view, pure Sb NPs provide lower losses at the LSPR frequency compared to the As${}_{1-z}$Sb${}_{z}$ alloy and pure As. A comparison of the data in Figure 3, Figure 4, Figure 5 and Figure 6 reveals the origin of this phenomenon—the dominant peak in $\epsilon_{2}$ for pure Sb has the highest strength, but it is shifted to the lowest energy of 1.85 eV. As a result, the LSPR in Sb–Al${}_{x}$Ga${}_{1-x}$As${}_{1-y}$Sb${}_{y}$ occurs with moderate values of $\epsilon_{2}$ between 10 and 15. This value is substantially larger than that for silver, the best plasmonic material for low-loss applications [49]. However, in contrast to As${}_{1-z}$Sb${}_{z}$ NPs, it is hardly possible to create an appropriate system of Ag NPs in the bulk of III-V semiconductors due to technological reasons.

## 5. Conclusions

Al${}_{x}$Ga${}_{1-x}$As and its related semiconductor compounds are basic materials for a wide variety of electronic, opto-electronic, and photonic devices. Our investigation of Sb-rich As${}_{1-z}$Sb${}_{z}$ nanoinclusions can expand the functionality of these widely used materials. We have discovered the origin of the plasmonic behavior of the metamaterial composed of the Sb-rich nanoparticles in the Al${}_{x}$Ga${}_{1-x}$As${}_{1-y}$Sb${}_{y}$ matrix. We show that the distribution of these nanoparticles in the matrix gives rise to the localized surface plasmon resonance near 2 eV. Our analysis is based on the ab initio calculations of the band structure and the dielectric susceptibility of As, Sb, and the As${}_{1-z}$Sb${}_{z}$ alloy, followed by the calculation of the optical extinction in terms of the Mie theory. The calculated optical spectra are well consistent with the available experimental data. The most important result of the calculations is that systems of Sb-rich nanoparticles can provide a resonant optical response near the edge of the band gap. The observation of the resonance in optical spectra is possible only when the semiconductor matrix has a sufficiently large band gap, which can be obtained with high concentrations of Al *x* and low concentrations of Sb *y* in the solid solution. At a high Al concentration *x* in the matrix and a high Sb concentration *z* in the As${}_{1-z}$Sb${}_{z}$ nanoparticles, the LSP resonance occurs near the semiconductor band gap edge, so it can substantially modify the linear and non-linear optical properties of the medium.

## Data Availability

The data that support the results of this study are available from the corresponding author upon reasonable request.

## Abbreviations

The following abbreviations are used in this manuscript:

LSP	Localized surface plasmon
LSPR	Localized surface plasmon resonance
NP	Nanoparticle
MBE	Molecular-beam epitaxy
VPE	Vapor-phase epitaxy
LT	Low temperature
BZ	Brillouin zone
TD-DFT	Time-dependent density functional theory
RPA	Random phase approximation
ALDA	Adiabatic local density approximation
PAW	Projector augmented wave
DP	Dirac point
